# Dynamic Balance between PTH1R-Dependent Signal Cascades Determines Its Pro- or Anti-Osteogenic Effects on MSC

**DOI:** 10.3390/cells11213519

**Published:** 2022-11-07

**Authors:** Konstantin Kulebyakin, Pyotr Tyurin-Kuzmin, Leila Sozaeva, Nikita Voloshin, Mikhail Nikolaev, Vadim Chechekhin, Maxim Vigovskiy, Veronika Sysoeva, Elizaveta Korchagina, Daria Naida, Maria Vorontsova

**Affiliations:** 1 Faculty of Medicine, Lomonosov Moscow State University, 119991 Moscow, Russia; 2 Institute for Regenerative Medicine, Medical Research and Education Center, Lomonosov Moscow State University, 119234 Moscow, Russia; 3 Endocrinology Research Center, 115478 Moscow, Russia; 4 Burdenko Main Military Clinical Hospital, 105094 Moscow, Russia

**Keywords:** parathyroid hormone, mesenchymal stromal cells, osteogenesis, calcium signaling

## Abstract

Parathyroid hormone (PTH) is one of the key regulators of calcium and phosphate metabolism in the body, controlling bone metabolism and ion excretion by the kidneys. At present, attempts to use PTH as a therapeutic agent have been associated with side-effects, the nature of which is not always clear and predictable. In addition, it is known that in vivo impairment of PTH post-receptor signaling is associated with atypical differentiation behavior not only of bone cells, but also of connective tissues, including adipose tissue. In this work, we studied the functional responses of multipotent mesenchymal stromal cells (MSCs) to the action of PTH at the level of single cells. We used MSCs isolated from the periosteum and subcutaneous adipose tissue to compare characteristics of cell responses to PTH. We found that the hormone can activate three key responses via its receptor located on the surface of MSCs: single transients of calcium, calcium oscillations, and hormone-activated smooth increase in intracellular calcium. These types of calcium responses led to principally different cellular responses of MSCs. The cAMP-dependent smooth increase of intracellular calcium was associated with pro-osteogenic action of PTH, whereas phospholipase C dependent calcium oscillations led to a decrease in osteogenic differentiation intensity. Different variants of calcium responses are in dynamic equilibrium. Suppression of one type of response leads to increased activation of another type and, accordingly, to a change in the effect of PTH on cell differentiation.

## 1. Introduction

Bone tissue is a dynamic structure that is intensively renewed throughout an individual’s life. The dynamic balance between resorption and synthesis plays a key role in the renewal and remodeling of bone tissue. Bone resorption is provided by cells of monocytic origin—osteoclasts. On the other hand, bone tissue renewal is provided by cells of mesenchymal origin—osteoblasts, the pool of which is replenished due to the differentiation of multipotent mesenchymal stromal cells (MSCs), located mainly in endosteum and periosteum and sometimes termed as skeletal stem cells [1]. Disruption in bone remodeling processes is a most important pathogenetic link in the development of several diseases such as osteoporosis, McCune–Albright syndrome, and Paget’s disease.

Endocrine and paracrine signals that regulate the activity of osteoclasts and osteoblasts play a leading role in maintaining the balance of resorption/laying of bone tissue. A major hormone that regulates bone remodeling and metabolism is the parathyroid hormone (PTH), which is secreted by the cells of the parathyroid glands. PTH is an 84 amino acid peptide hormone involved in the control of calcium homeostasis [2]. Acting on the kidneys, bone tissue, and intestine, it provides an increase in blood levels of Ca^2+^ ions and a decrease of phosphate. At the same time, acting on the bone tissue, PTH can promote differentiation of osteoblasts and bone formation [3,4]. Its pro-osteogenic effects are mediated by activation of the Wnt/β-catenin signaling pathway [5] and by an increase in the effectiveness of BMP signaling—the main morphogenic stimulus responsible for differentiation of osteoblasts from MSCs [6]. Due to its effect on bone metabolism, PTH is viewed as a therapeutic agent for osteoporosis [7], fractures [8], and a number of other disorders of bone renewal and regeneration [3,4]. At the same time, the use of PTH in clinical practice is significantly limited by the fact that PTH was shown to cause complex, sometimes contradictory effects in bone tissue [9]. This is due to the presence of several groups of cells that react differently to this hormone. One the one hand, PTH has a direct effect on osteocytes and osteoblasts, triggering the processes of osteogenesis. In addition, it regulates the renewal of bone cells by acting on resident MSCs. On the other hand, PTH indirectly promotes bone resorption by activating osteoclasts via the PTH-induced paracrine action of osteoblasts [10,11].

From clinical practice, it is known that, with a constant administration of PTH, there is an intensification of bone resorption and calcium mobilization from bone tissue, while an intermittent administration of PTH leads to bone formation activation [12]. Nevertheless, this type of therapy has a number of side-effects and a frequently unpredictable withdrawal effect. In addition, it is known that, in some pathologies with permanently elevated blood levels of PTH and impaired mechanisms of intracellular signaling (for example, pseudohypoparathyroidism), foci of ectopic ossification and calcifications in adipose tissue are observed [13]. It is assumed that the occurrence of these foci is a consequence of an incorrect differentiation response of adipocyte MSCs to the excessive action of PTH [14].

The cellular effects of PTH in the adult organism are determined mainly by it binding to the PTH type 1 receptor (PTH1R), which belongs to the group of G-protein coupled receptors (GPCR) class B [15]. PTH1R is expressed in various tissues and organs such as bone, kidney, adipose tissue, and intestine. The second isoform of the PTH receptor, PTH2R, plays a key role in embryonic development. In the adult body, its expression decreases significantly, and the receptor can be found in the nervous tissue and some other highly specialized tissues of the human body [16]. PTH1R can activate both the trimeric Gs protein, triggering intracellular cAMP synthesis, and the Gq protein, coupled to the inositol-1,4,5-phosphate/Ca^2+^ signaling cascade [17]. In recent years, many studies provided insight, showing that these signaling cascades may be responsible for the implementation of fundamentally different cellular effects of PTH [4].

We demonstrate that MSCs isolated from the periosteum and adipose tissue differ in their ratio of cells that respond to PTH by one or the other form of calcium response. The predominant calcium response of MSCs to PTH determines the direction of the influence of this hormone on osteogenic differentiation. The cAMP-dependent gradual increase in calcium is associated with the pro-osteogenic PTH action, while calcium oscillations mediate the anti-osteogenic PTH action. We show that these signaling cascades are in a dynamic equilibrium, whereby suppression of one of the signaling cascades leads to the activation of the opposite one in the same cells. This opens prospects for overcoming the problems of using PTH for bone regeneration and the treatment of osteoporosis, associated with the multiplicity of effects of this hormone on resident bone tissue stem cells.

## 2. Materials and Methods

### 2.1. MSC Isolation and Culturing

MSCs were isolated from subcutaneous fat tissue or periosteum of 16 healthy young donors using enzymatic digestion as previously described [18]. The average donor’s BMI was 23.5 ± 2.4, age 45.25 ± 4.11 years, and none had symptoms of acute inflammation, diabetes, or oncological diseases. Abdominal subcutaneous fat tissue was obtained during abdominal surgery; the periosteum was obtained during arthroscopic surgery of the knee. All procedures performed with tissue samples from patients were in accordance with the Declaration of Helsinki and approved by the Ethic Committee of Burdenko Main Military Clinical Hospital (Moscow, Russia), protocol #160 (date of approval 22 July 2019). All donors provided informed consent. Cells were cultured in AdvanceSTEM Mesenchymal Stem Cell Media containing 10% AdvanceSTEM Supplement (HyClone, Cytiva, Marlborough, MA, USA), 1% antibiotic–antimycotic solution (HyClone, Cytiva, Marlborough, MA, USA), and 1% L-glutamine (Gibco, Thermo Fisher Scientific, Waltham, MA, USA) at 37 °C in a 5% CO_2_ incubator (Binder, Tuttlingen, Germany, CB210). Cells were passaged at 80–90% confluency using Versen solution (Paneco, Moscow, Russia) and HyQTase solution (HyClone, Cytiva, MA, USA). For the experiments, MSCs cultured up to 5–6 passages were used. To confirm their multipotency, MSCs were induced into osteogenic, adipogenic, and chondrogenic differentiation as described earlier [18].

### 2.2. Flow Cytometry

MSC immunophenotype was analyzed using flow cytometry. After medium harvesting, cells were detached from culture dishes using Versene solution and stained with antibodies against CD73, CD90, and CD105 (MSC Phenotyping Kit, Miltenyi Biotec, Bergisch Gladbach, Germany) as described in the manufacturer’s protocol. IgGs of appropriate isotype were used as a negative control. Stained cells were analyzed using FACS ARIA III cell sorter (BD, Franklin Lakes, NJ, USA).

### 2.3. MSCs Differentiation Assays

The ability of MSCs to differentiate into the osteogenic direction was tested in vitro using standard differentiation and analysis protocols described previously [18]. Briefly, cells were cultured in 12-well culture plates up to 100% confluence in all differentiation experiments. Osteogenic differentiation was induced by incubating MSCs on collagen- and vitronectin-covered plates in a growth medium containing StemPro Osteogenesis Differentiation Kit (Thermo Fisher Scientific, Waltham, MA, USA) for up to 21 days. The medium was changed every 2 days. For inhibitor analysis, PLC and AC inhibitors (U73122 (Sigma-Aldrich, St. Louis, MO, USA) and SQ22536 (Sigma-Aldrich, St. Louis, MO, USA)) were added 1 h prior to the addition of PTH. Differentiation efficiency was analyzed using Alizarin Red S staining.

### 2.4. Histologic Staining

Alizarin Red was used to estimate the efficiency of osteogenic differentiation. Cells were fixed with 10% paraformaldehyde for 30 min. Then, they were stained with the Osteogenesis Quantitation Kit (Millipore, Burlington, MA, USA) according to the manufacturer’s instructions. Cell cultures were stained with Nile Red (Sigma-Aldrich, St. Louis, MO, USA) to determine the efficiency of adipogenic differentiation. Cells were washed with HBSS with 20 nM HEPES and then stained for 1 h in a cell culture incubator in HBSS with 20 nM HEPES containing 1 mM of Nile Red. Obtained samples were analyzed via Nikon Eclipse Ti2 microscope with a Kinetix camera (Teledyne Photometrics, Tucson, AZ, USA). Image capture and analysis was performed using NIS Elements AR 5.40.02 software.

### 2.5. RT-PCR

RNeasy Mini Kit (Qiagen, Hilden, Germany) was used to extract RNA. cDNA was synthesized from 500 ng RNA with MMLV Reverse Transcription Kit (Evrogen, Moscow Russia) according to the manufacturer’s instructions. The relative expression of gene markers of osteogenic differentiation (*RUNX2*, *OCN*) was analyzed using quantitative real-time PCR. The following equipment was used: qPCR mix-HS SYBR + LowROX (Evrogen, Moscow, Russia) reagents and CFX96 Touch Real-Time PCR Detection System (Bio-Rad). The gene of the 60S Ribosomal protein P0 (*RPLP0*) was used as a housekeeping gene. Quantification and normalization of expression levels of both the target and the reference genes (*RPLP0*) were calculated using the comparative threshold cycle (CT) method. Primers for PCR were picked using the NCBI Primer Designing Tool. Primer sequences are provided in Table 1.

### 2.6. Calcium Imaging

PTH1R activation was assessed using PTH (1–34) and the Ca^2+^ imaging technique described earlier [19]. Cells grown in HyClone Advance Stem medium with Supplement in 48 well plates were loaded with Fluo-8 (Abcam, Cambrige, UK, ab142773, 4 µM), in Hanks Balanced Salt Solution with 20 mM Hepes, for 1 h. Cells were grown at low density to prevent cell-to-cell communications during the calcium imaging. To analyze the pattern of calcium responses, we recorded the baseline for 10–20 min, and then added PTH without stopping the recording. To evaluate the role of cAMP-dependent signaling or phospholipase C (PLC)-dependent signaling in the realization of different patterns of calcium signaling, we stimulated cells with either noncompetitive adenylate cyclase inhibitor SQ22536 (Abcam, ab120642, 1 µM) or PLC inhibitor U73122 (Abcam, Cambrige, UK, Cat# ab120998, 1 µM). Cells were loaded with Fluo-8 for 1 h before the experiment, and then inhibitors were added 30 min before the experiment. Intracellular Ca^2+^ responses were measured in individual cells using an inverted fluorescent microscope Nikon Eclipse Ti2 equipped with an objective CFI Plan Fluor DLL 10 × 0.3 (Nikon, Tokyo, Japan) and with a digital EMCCD camera Andor iXon 897 (Andor Technology, Belfast, UK). We used the simultaneous measuring of 3 × 3 fields of view in large image mode to increase the number of analyzed cells. Movies were analyzed using NIS-Elements (Nikon, Tokyo, Japan) and ImageJ software. Alterations of cytosolic Ca^2+^ from the resting level were quantified by relative changes in the intensity of Fluo-8 fluorescence (ΔF/F0) recorded from an individual cell. The percentage of cells responding was measured as the ratio of the number of cells responding to the number of all analyzed cells in 3 × 3 fields of view. To analyze the switching of the format of calcium response after inhibition of AC- or PLC-dependent signaling, we used repetitive addition of PTH to the same cells. At first, adipose-derived MSCs or periosteum-derived MSCs were stimulated with PTH under control conditions. Then, 40 min after recording the responses, the cells were washed three times with three changes of the medium in 5 min intervals. Then, the cells were then incubated with inhibitors SQ22536 or U73122 for 15 min, and the cells were again stimulated with PTH in the presence of the inhibitor.

## 3. Results

### 3.1. MSCs from Periosteum and Adipose Tissue Contain a Comparable Number of PTH1R-Positive Cells

**Figure 1:**
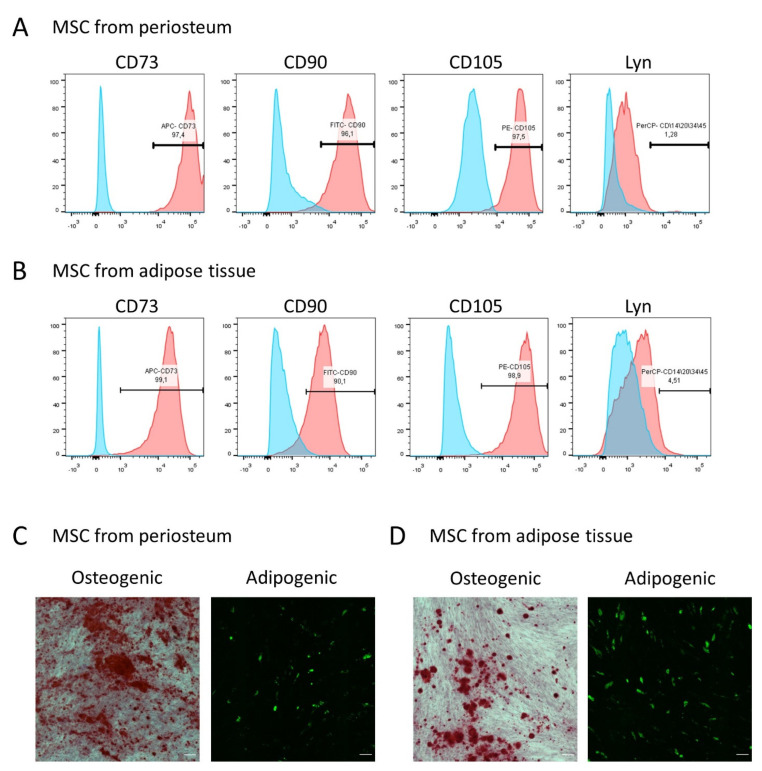
Characterization of MSC isolated from periosteum and adipose tissue. (**A**,**B**) Flow cytometry analysis of expression of positive surface markers CD73, CD90, and CD105 and negative marker Lyn on MSC from periosteum (**A**) and adipose tissue (**B**). (**C**,**D**) Differentiation of MSC from periosteum (**C**) and adipose tissue (**D**) into osteogenic (Alizarin red staining) and adipogenic lineage (Nile red staining). The scale bar represents 500 μm.

To analyze the functional responses of MSCs to PTH exposure, these cells were isolated from two tissue depots: subcutaneous adipose tissue of the abdomen and periosteum of the tibia and/or ilium. As can be seen from Figure 1, all these cells had an immunophenotype characteristic of MSCs. Most of the cells in the populations expressed surface CD73, CD90, and CD105 (Figure 1A,B) and were capable of differentiating into typical directions for MSC [20]—adipogenic and osteogenic (Figure 1C,D).

**Figure 2:**
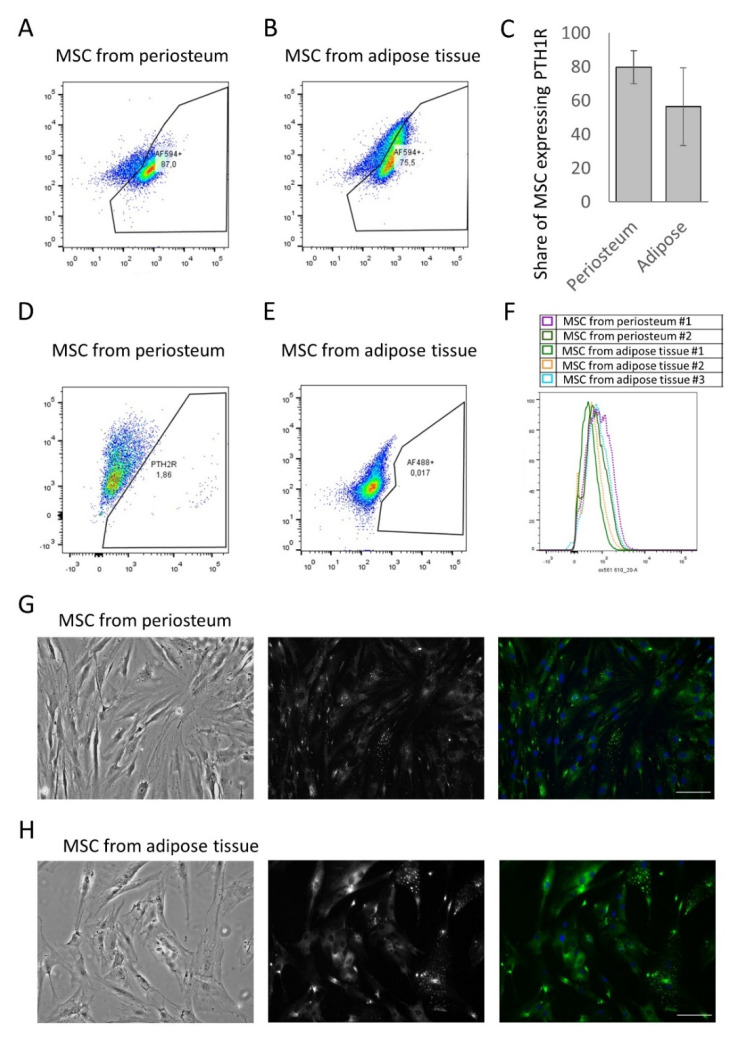
Analysis of PTH1R and PTH2R expression in MSC isolated from periosteum and adipose tissue. (**A**,**B**) Representative flow cytometry graphs of PTH1R expression in MSC isolated from periosteum (**A**) and adipose tissue (**B**). (**C**) Statistical analysis of PTH1R expression measured by flow cytometry. Mean ± SE, n = 3–4 different donors. (**D**,**E**) Representative flow cytometry graphs of PTH2R expression in MSC isolated from periosteum (**D**) and adipose tissue (**E**). (**F**) Flow cytometry analysis of PTH1R density on the cell surface of MSC from periosteum and adipose tissue. (**G**,**H**) Representative immunofluorescent images of PTH1R expression in MSC isolated from periosteum (**G**) and adipose tissue (**H**) The scale bar represents 500 μm.

At the first stage, an analysis was carried out for the expression of PTH1R and PTH2R on the surface of MSCs isolated from the two depots mentioned above. Analysis using flow cytometry showed that most MSCs of both the periosteum and the adipose tissue expressed the PTH1R receptor on their surface, but not PTH2R (Figure 2A–E). The share of periosteum MSCs expressing PTH1R was 78.4 ± 11.3%, whereas, in the population of adipose tissue, PTH1R was expressed in 69.4 ± 16.1% of MSCs (Figure 2C). PTH2R was not detected using flow cytometry (Figure 2D,E). Specific expression of PTH1R at the cell surface was confirmed using immunofluorescence microscopy (Figure 2G,H). Thus, MSCs from the periosteum and adipose tissues contained a similar number of PTH1R-positive cells. Remarkably, in the case of PTH1R, it has been reported that the density of receptors on the cell surface may affect the downstream signaling profile [21,22]. To assess the receptor density on MSC from periosteum and adipose tissue, we compared the intensity of fluorescent signal in flow cytometry experiments. Our data indicated no difference in the relative amount of PTH1R per cell in MSC from different depots (Figure 2F).

### 3.2. Parathyroid Hormone (PTH) Has a Different Effect on the Differentiation of MSCs Derived from Periosteum and Adipose Tissue

**Figure 3:**
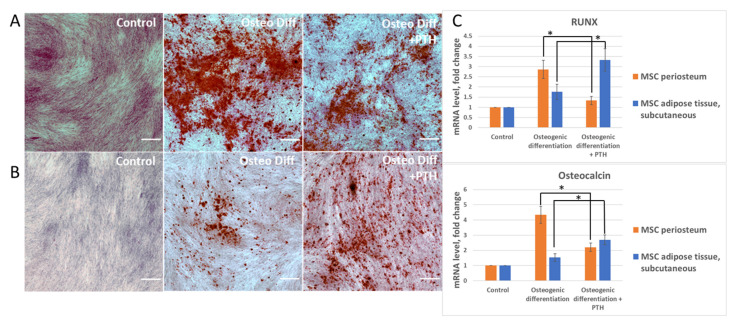
Periosteum MSC and adipose tissue MSC demonstrate different responses to PTH stimulation during osteogenesis. All presented pictures of histochemical staining were obtained on the cells from different MSC depots of the same donor. (**A**,**B**) Representative images of osteogenic differentiation of MSC from periosteum (**A**) and from adipose tissue (**B**) obtained from the same donor with or without stimulation by PTH. Pictures were taken after 14 days of osteogenic induction. The scale bar represents 500 μm. (**C**) Expression of osteogenic markers in MSC on 14th day of osteogenesis. Mean ± SE, n = 4 different donors; * *p* < 0.05 versus corresponding values in the “osteogenic differentiation” group.

The effect of PTH treatment on the osteogenic differentiation of MSCs isolated from the periosteum and adipose tissues was tested. As mentioned earlier, PTH can demonstrate different effects on different donors. To negate this effect, we obtained both periosteum and adipose tissue from each donor. All comparison experiments between periosteum and adipose tissue were performed on the cells obtained from the same donor. MSCs osteogenic differentiation was induced both in the absence and in the presence of PTH (10 nM). We found that PTH affected the osteogenic differentiation of MSCs derived from those two tissue depots in different ways. As shown in Figure 3A,C, the presence of PTH in the osteogenic medium of periosteal MSCs led to a significant reduction in differentiation efficiency when compared to control conditions. At the same time, PTH had a pronounced pro-osteogenic effect on MSCs obtained from the adipose tissue of the abdomen. Specifically, both an increase in calcium deposits, stained with Alizarin red, and an increase in the expression of osteogenesis marker genes were recorded in comparison with the group of cells which underwent differentiation under control conditions (Figure 3B,C). Comparing the expression levels of gene-markers of osteogenic differentiation showed that, despite the initially lower potential for osteogenic differentiation in the adipose MSCs, PTH enhances this process to values similar to those in periosteum MSCs. Thus, the assumption may be made that PTH has a multidirectional effect on the osteogenic differentiation of MSCs isolated from various tissue depots.

### 3.3. MSCs Derived from Different Tissue Depots Show a Different Profile of Calcium Signals in Response to PTH

To answer the reason for the multidirectional effect of PTH on the osteogenic differentiation of MSCs isolated from various tissue depots, the signaling activity of these cells in response to hormone stimulation was studied. We recorded PTH-activated intracellular calcium signaling at the single-cell level using intracellular calcium dye Fluo-8.

**Figure 4:**
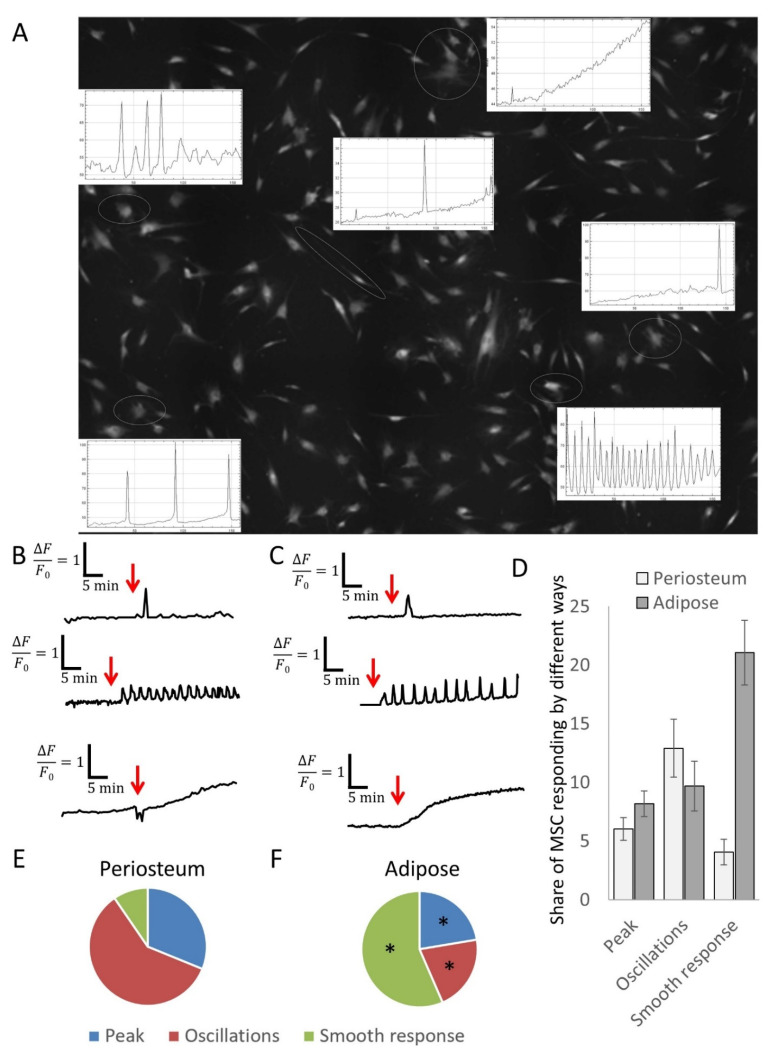
Calcium signaling in MSCs derived from adipose tissue and periosteum. (**A**) Representative snapshot of MSCs stained with Fluo-8 dye with graphs of responses of the single cells. Individual cells of the same population of adipose tissue (**B**) and periosteum (**C**) responded in one of three ways: single calcium peak, calcium oscillations, or a smooth increase in intracellular calcium level. (**D**) Number of adipose-derived and periosteum-derived MSCs responding in the different ways. (**E**,**F**) Share of MSCs responded in the different ways among all responding cells in MSCs from adipose tissue (**E**) and periosteum (**F**). Mean ± SE, n = 6–7 different donors; * *p* < 0.05 versus corresponding periosteum values.

Three main variants of the response of single MSCs to PTH exposure were identified. The first type of response was a single transient increase in the intracellular calcium level (Figure 4A, Supplementary Video S1), the amplitude of which did not depend on the actual hormone concentration. This response, as we previously showed for MSCs, indicates the involvement of the calcium-induced calcium release mechanism (CICR) [23]. The second variant of recorded response was calcium oscillations. These were realized in two ways; in the first, oscillations appeared after the action of PTH on cells, whereas, in the second, the cells oscillated even in the absence of the hormone, but exposure to PTH increased the frequency of oscillations (Figure 4A–C). The third type of MSC responses we recorded was a gradual increase in intracellular calcium level, lasting from 20 min to 60 min or more (Figure 4A–C). Thus, single MSCs demonstrated several different patterns of calcium responses to PTH, which may underlie differences in the effect of PTH on osteogenic differentiation of MSCs isolated from periosteum and adipose tissue.

The analysis showed that cell cultures obtained from periosteum and adipose tissue differed significantly in the representation of various variants of calcium responses. As seen in Figure 4D–F, the proportion of cells responding to each of the response options varied significantly. This suggests that the physiological effects of PTH on MSCs from different depots may be due to the activation of different signaling pathways.

### 3.4. Inhibitory Analysis Demonstrates the Possibility of Switching the Types of Ca^2+^ Responses to PTH in MSC Populations

**Figure 5:**
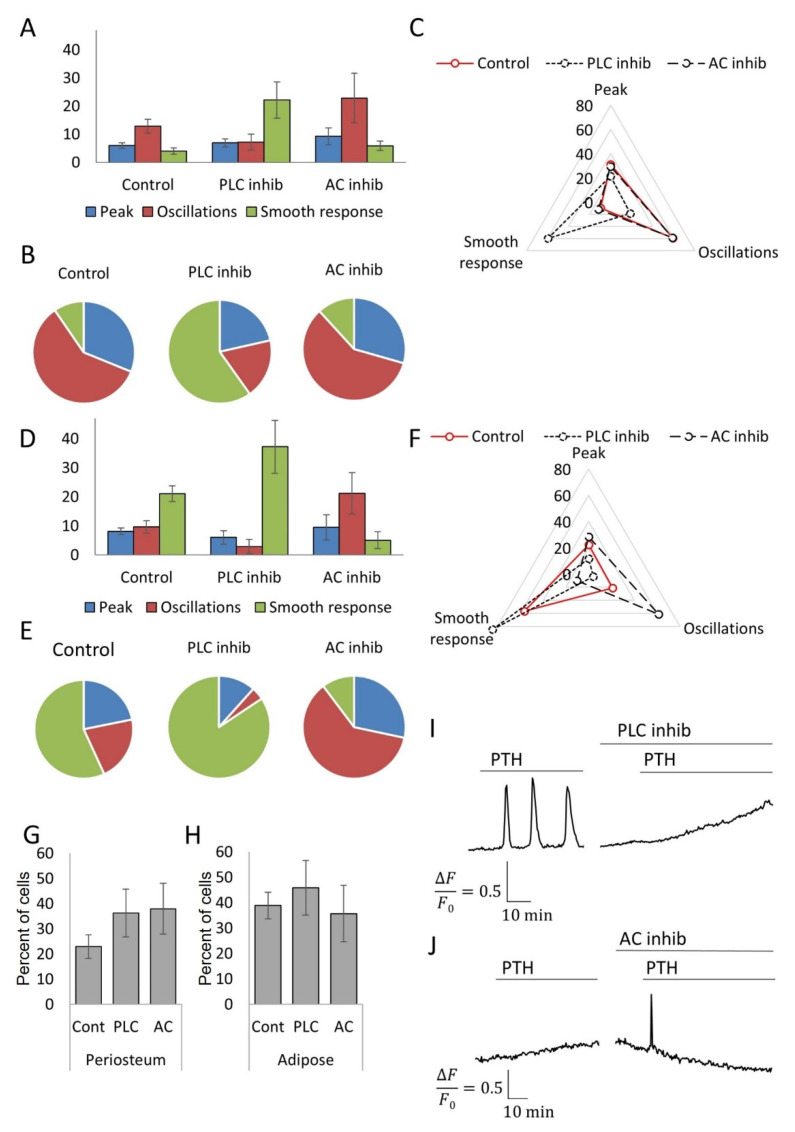
Inhibitory analysis of adipose-derived and periosteum-derived MSCs. (**A**–**C**) Influence of inhibition of PLC and AC on adipose-derived MSC responding to PTH. The number (**A**) and share (**B**) of adipose-derived MSC responding in the different ways of calcium response. (**C**) Radar chart of changes in the share of adipose-derived MSC responding in the different ways of calcium response. (**D**–**F**) Influence of inhibition of PLC and AC on periosteum-derived MSC responding to PTH. The number (**D**) and share (**E**) of periosteum-derived MSC responding in the different ways of calcium response. (**F**) Radar chart of changes in the share of periosteum-derived MSC responding in the different ways of calcium response. (**G**,**H**) Total number of MSCs responding to PTH in any way of calcium response did not change significantly. (**G**) Percentage of adipose-dependent MSCs responding to PTH. (**H**) Percentage of periosteum-dependent MSCs responding to PTH. (**I**,**J**) Individual cells dynamically switched their calcium responses to PTH under the action of inhibitors. (**I**) Representative graph of PTH-induced calcium level changes in the single cell in adipose-derived MSCs population in the control conditions and after AC inhibition. (**J**) Representative graph of PTH-induced calcium level changes in the single cell in periosteum-derived MSCs population in the control conditions and after PLC inhibition. In all experiments, PTH was added in a concentration of 10 nM; the phospholipase C (PLC) inhibitor is U73122 (1 μM), while the adenylate cyclase (AC) inhibitor is SQ22536 (1 μM).

It is known that the parathyroid hormone receptor can interact with two different G-proteins for signal transmission into the cell: the G_s_ protein, which activates adenylate cyclase and protein kinase A, and the G_q_ protein, which activates phospholipase C and IP3-dependent calcium release [24]. To establish the contribution of these two signaling pathways to the realization of the specific pattern of calcium response to PTH in MSCs, we used selective inhibitors of phospholipase C (U73122) and adenylate cyclase (SQ22536). We found that the use of inhibitors led to a change in the repertoire of cellular responses to PTH in MSCs obtained from periosteum and adipose tissue. Inhibition of adenylate cyclase led to a decrease in the number of cells responding with a gradual increase in Ca^2+^ levels, while simultaneously increasing the number of cells responding to PTH with Ca^2+^ oscillations or a single peak increase (Figure 5A–F). On the other hand, a phospholipase C inhibitor reduced the number of cells responding with Ca^2+^ oscillations but increased the number of cells responding with a gradual increase in Ca^2+^ (Figure 5A–F). It is noteworthy that the inhibition of individual signaling cascades did not lead to a decrease in the total number of cells in the population responding to PTH but led to a redistribution of Ca^2+^ response types (Figure 5G,H).

We investigated the reason for the change in the predominant format of the response of cells in the population upon inhibition of AC or PLC is. It can be mediated by a change in the population composition of responding cells, or by the switching of the signaling pathway in the individual cells. To answer this question, we measured the responses of the same single cells in the before and after the addition of inhibitors. As shown in Figure 5I, oscillatory response of periosteum MSC switched to smooth increase in intracellular Ca^2+^ level in the same cell as a result of PLC inhibition. Figure 5J shows an example of adipose-derived MSC in which a gradual increase in Ca^2+^ level changed to a single transient calcium response. Thus, PTH activated a different repertoire of calcium-dependent responses in the periosteum and adipose-derived MSCs, and a particular format of Ca^2+^ response could be switched at the level of individual cells.

### 3.5. Switching of Intracellular Ca^2+^ Signaling Makes It Possible to Change the Vector of PTH Influence on the Osteogenic Differentiation of MSCs

Changes in the repertoire of Ca^2+^ responses to PTH in MSCs upon inhibition of adenylate cyclase and phospholipase C may lead to modulation of the pro-/anti-osteogenic effect of PTH on MSCs derived from various tissue depots. To test this hypothesis, we induced osteogenic differentiation of periosteum- and adipose-derived MSCs in the presence of AC and PLC inhibitors.

**Figure 6:**
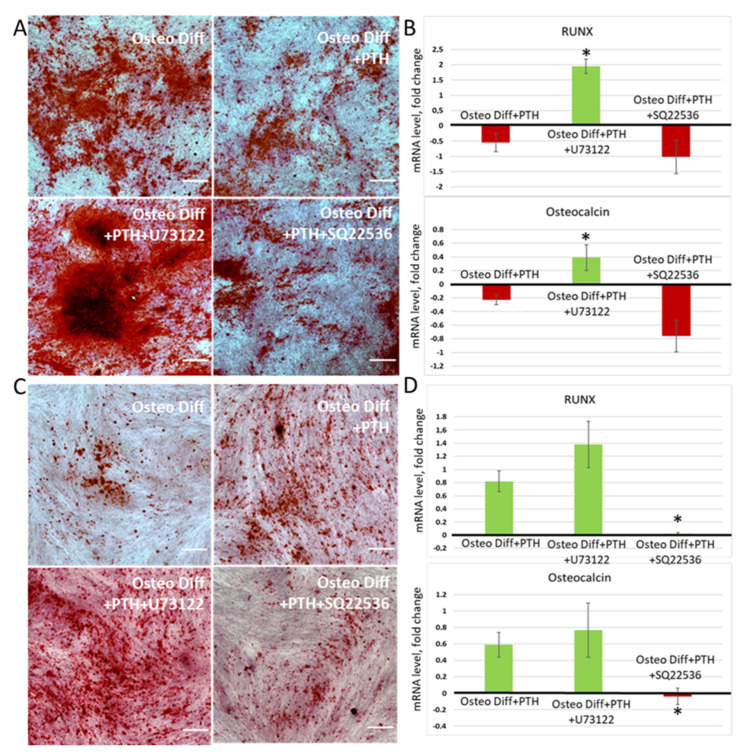
PLC (U73122) and AC (SQ22536) inhibitors modulate the effect of PTH on MSC osteogenic differentiation. All presented pictures of histochemical staining were obtained on the cells from different MSC depots of the same donor (**A**,**B**) Differentiation of MSC from periosteum in presence of PTH and inhibitors of PTH1R-related secondary messenger systems. (**A**) Histochemical staining of calcium deposits with Alizarin red. The scale bar represents 500 μm. (**B**) Expression of osteogenic markers in MSC from periosteum on 14th day of osteogenesis. Mean ± SE, n = 4 different donors; * *p* < 0.05 versus values in “osteogenic differentiation + PTH” group. (**C**,**D**) Differentiation of MSC from adipose tissue in presence of PTH and inhibitors of PTH1R-related secondary messenger systems. (**C**) Histochemical staining of calcium deposits with Alizarin red. The scale bar represents 500 μm. (**D**) Expression of osteogenic markers in MSC from adipose tissue on 14th day of osteogenesis. Mean ± SE, n = 4 different donors; * *p* < 0.05 versus values in “osteogenic differentiation + PTH” group.

As can be seen from Figure 6, in both cases, preincubation of cells with a PLC inhibitor led to an increase in osteogenic differentiation in the presence of PTH. Note that, in cells obtained from the periosteum, the inhibitor completely changed the effect of PTH on osteogenesis; instead of an anti-osteogenic effect, the hormone began to have a pro-osteogenic effect (Figure 6A,B). The AC inhibitor, in turn, potentiated the anti-osteogenic effect of PTH on cells from the periosteum and completely leveled the pro-osteogenic effect of PTH on cells from the subcutaneous adipose tissue (Figure 6C). Thus, it can be concluded from the obtained results that, in MSCs, the key signaling mechanism responsible for the pro-osteogenic effect of PTH is a cAMP-dependent gradual increase in the intracellular Ca^2+^ level. Phospholipase C-induced Ca^2+^ oscillations, in turn, mediate the anti-osteogenic effect of PTH on MSCs. It is likely that PTH1R in MSC cells is in dynamic interaction with both phosphoinositide-dependent and cAMP-dependent signaling cascades, and the prioritization of one or the other pathway determines the functional effect of PTH on cells. This dynamic balance can be shifted at the level of single cells when using selective inhibitors of signaling cascades, which leads to a change in the PTH effect on osteogenesis.

## 4. Discussion

The effect of PTH on the connective tissues of the body is largely controversial. For example, the intermittent action of PTH activates bone formation, while the constant introduction of PTH enhances the process of bone resorption and suppress osteogenesis. In hereditary diseases associated with impaired PTH receptor signaling, such as pseudohypoparathyroidism or progressive osteoid heteroplasia, foci of ectopic ossification or ectopic bone formation occur in the subcutaneous adipose tissue, respectively [25,26,27]. This phenomenon may be based on the heterogeneity of MSC responses in adipose and bone tissues.

The heterogeneity of hormonal responses of MSCs from different tissue depots can be associated either with different expression of PTH receptors or with different activated signaling cascades from the same receptor. After analyzing the expression of PTH receptors, we showed that both periosteal MSCs and adipose tissue MSCs almost exclusively express PTH1R. PTH2R is expressed in 1–2% of periosteal cells and is unlikely to make a significant contribution to the signaling response of the entire MSC population. We showed at the single-cell level that individual cells respond heterogeneously to PTH. MSCs can form three different calcium-dependent signaling responses: phosphoinositide-dependent single peaks, calcium oscillations, and a cAMP-dependent gradual increase in intracellular calcium levels. Each individual cell responds with one of the listed types of response. Notably, early studies of Ca^2+^ signaling showed that osteoblasts respond to PTH in a biphasic mode. First, a short IP_3_-dependent calcium peak was recorded, which was replaced by a smooth cAMP-dependent increase in the level of intracellular calcium [28]. Most likely, these results were obtained on the total cell population and are a product of overlay of three types of recorded responses to PTH, which can be differentiated using a single-cell approach. Thus, PTH1R activates three alternative signal cascades in MSCs from both periosteum and adipose tissue; however, the percentage of MSCs responding with each individual pattern greatly differs in these depots.

There are multiple reasons for individual hormones activating the same receptor causing different signaling responses in different cells. Primarily, individual cells can demonstrate different expression patterns of the GPCR signaling partners and, thus, respond in a unique way to the PTH, as it has been reported for other receptors [29]. However, we demonstrated a dynamic manner of interchange between different calcium signaling patterns in MSC. The change in signaling was observed within 1 h after inhibition of specific signaling pathway. This excludes the possibility of expression heterogeneity and suggest that signaling partners for both PLC- and AC-dependent signaling pathways are presented in MSCs in a state of dynamic competition. While there are data suggesting that the choice between PLC- and AC-dependent signaling may be associated with the density of PTH1R on cell surface [30], we observed no difference in MSCs from the periosteum and adipose tissue. Therefore, the observed dynamic state may be realized through the activity of adaptor proteins, namely, NHERF, which is known to participate in the switching of PTR1R signaling to the IP3-dependent pathway [30]. Another possible mechanism of upholding the dynamic competition includes interaction between downstream effectors of those signaling pathways. For example, involvement of the isoform of AC inhibitable by calcium (ADCY7) [31] makes calcium-dependent signaling antagonistic to cAMP-dependent signaling.

This study showed that PTH affects MSCs derived from various tissues in different ways. In the case of MSCs from adipose tissue, PTH showed a pronounced pro-osteogenic effect, while, in the case of periosteum MSCs, exposure to PTH led to a decrease in the ability to undergo osteogenic differentiation. It should be noted that this study is limited by the fact that cells were incubated in rather high concentrations of PTH before differentiation was initiated, without the possibility of mimicking the changes in PTH levels that occur in the body. Thus, the ability to undergo osteogenic differentiation or patterns of responses to PTH may differ depending on PTH concentrations. Nevertheless, our results demonstrate that the response to PTH differs significantly in MSCs of different localization in the body. In addition, the study showed that the outcome of PTH-modulated osteogenic differentiation of MSCs can change depending on which intracellular signaling pathway is blocked. Thus, blocking the phospholipase C pathway leads to a pronounced increase in the osteogenic potential of MSC cells in response to PTH, whereas blocking the adenylate cyclase pathway leads to a sharp decrease in this potential.

At the same time, the data from clinical practice suggest that blocking the Gαs signaling pathway would lead to an increase in osteogenic differentiation. For example, patients with pseudohypoparathyroidism, which is associated with inactivating mutations in the *GNAS* gene encoding the alpha subunit of the G protein, often demonstrate foci of heterotopic ossification in subcutaneous fat [27,32]. At the same time, it was previously shown that the Gαs negatively regulates the Hedgehog signaling pathway and inhibits the ectopic ossification [33]. It is also known that, in the case of activating *GNAS* mutations (as in McCune–Albright syndrome), Wnt-β-catenin signaling is enhanced. This in turn contributes to impaired differentiation of osteoblasts in the bone, leading to fibrous tissue dysplasia [34].

It is worth noting that the development of heterotopic ossification has not been described in patients with acrodysostosis, despite the fact that, in this disease, protein kinase A is inhibited [32]—a protein involved in Gαs signaling. There is also no ectopic ossification in patients with PTH resistance associated with receptor gene mutations (Aiken syndrome and Bloomst chondrodysplasia) leading to the absence of any signaling following receptor activation [32].

Furthermore, at the molecular level, the opposite effect of the cAMP/PKA-dependent signaling cascade has been shown [4]. One of the effects of PTH in bone tissue is the activation of anabolic processes, which leads to an increase in the number of osteoblasts and an increase in their activity. The main signaling cascade that determines this anabolic action is Gαs/cAMP/PKA. PTH-dependent activation of PKA leads to phosphorylation of the cAMP response element-binding protein (CREB) transcription factor, which, in turn, interacts with the early response transcription factors c-jun and c-fos involved in the formation of the transcription initiation complex. Thus, a PTH-dependent increase in the expression of key genes responsible for osteogenic differentiation, such as Runx2, osteocalcin, and alkaline phosphatase, occurs [35]. Moreover, PKA can phosphorylate additional targets that provide osteogenesis genes expression. These targets include the transcription factor αNAC (nascent polypeptide-associated complex α-subunit), involved in PTH-dependent expression of osteocalcin [36], as well as mitogen-activated protein kinase p38, which has a part in the implementation of the anabolic action of PTH [37]. An important role in the implementation of the anabolic action of PTH is played by cross-activation of the Wnt/catenin-β signaling cascade, one of the main promoters of osteogenic differentiation of osteoblasts. Catenin-β is a target for PKA, and its phosphorylation leads to an increase in the stability of the molecule [38]. Simultaneously, PTHR1 dimerizes with the co-receptor Wnt/LRP6 (low-density lipoprotein receptor-related protein 6), which also leads to activation of the Wnt/catenin-β signaling pathway. In addition, PTH signaling suppresses the mechanism of inactivation of the discussed signaling cascade. PKA phosphorylates glycogen synthase kinase 3 (GSK3), which leads to downregulation of the latter. This disrupts the inhibitory complex composed from GSK3 and protein axin-2, which binds catenin-β and stops signal transduction along the Wnt/catenin-β pathway [5]. Finally, the action of PTH on osteoblasts leads to a decrease in the expression of sclerostin and Dickkopf 1 (Dkk1) proteins [38], both known inhibitors of canonical Wnt signaling and BMP signaling [39]. Enhancement of BMP signaling is another mechanism of PTH-mediated osteogenesis. PTH-dependent formation of the PTH1R/LRP6 signaling complex leads to an increase in BMP2-induced Smad1 phosphorylation, which in turn leads to the commitment of MSCs to the osteoblast differentiation [6].

Thus, various studies and examples of certain hereditary pathologies demonstrate that Gαs signaling can have multidirectional effects on the osteogenic differentiation of MSCs [40,41,42]. The exact mechanisms of PTH action and signaling pathways following receptor activation remain unclear. Obviously, this signaling is a subtle regulatory mechanism of MSC differentiation involving other signaling pathways (Hedgehog pathway and Wnt-β-catenin pathway) and multidirectional processes depending on the operating conditions of the receptor. According to the results of our study combined with literature data, the signaling pathway involving Gαs is of particular importance for osteogenic differentiation. Our study also demonstrated that the same signaling pathway can realize multidirectional effects depending on the tissue which the MSCs originated from.

In our work, we demonstrated, for the first time, that MSC signaling cascades associated with PTH1R may exist in dynamic competition, where downregulation of one may lead not to a decrease in responsiveness, but to a switch in signaling with a resulting outcome of osteogenesis. This result may herald a new possible strategy for altering the properties of stem cells used in tissue engineering and regenerative medicine.

The results of this study revealed some molecular features of MSC differentiation in the osteogenic direction under the action of PTH. Further studies of these features are needed, with the potential to reveal additional osteogenic regulatory mechanisms and new molecular targets. A possible investigation vector may be taken in the direction of studying adaptor proteins, like NHERF, which are part of the PTH1R/IP3 signaling pathway. Furthermore, downstream effectors, such as AC and its isoforms, may be studied. In particular, the calcium-inhibitable AC-7 isoform could be of interest. New therapy targets are necessary for a better, more effective treatment of various disorders of bone metabolism, including osteoporosis and other less common or orphan diseases, such as progressive osteoid heteroplasia, McCune–Albright syndrome, and pseudohypoparathyroidosis.

## Figures and Tables

**Table 1:** List of primers used in present work.

Target	Gene	Primers	Amplicon Size (bp)
60S Ribosomal protein P0	*RPLP0*	F: GCTGCTGCCCGTGCTGGTG R: TGGTGCCCCTGGAGATTTTAGTGG	130
Runt-related transcription factor 2	*RUNX2*	F: TCTTAGAACAAATTCTGCCCTTT R: TGCTTTGGTCTTGAAATCACA	136
Osteocalcin	*OCN*	F: AGCAAAGGTGCAGCCTTTGT R: GCGCCTGGGTCTCTTCACT	63

## Data Availability

Not Applicable.

## Supplementary Materials

The following supporting information can be downloaded at https://www.mdpi.com/article/10.3390/cells11213519/s1: Video S1: Representative PTH signaling.
